# Photonics of High-Entropy Polymers Revealing Molecular Dispersion via Polymer Mixing

**DOI:** 10.1021/acsnano.4c10585

**Published:** 2024-11-16

**Authors:** Yu-Jr Huang, Jien-Wei Yeh, Arnold Chang-Mou Yang

**Affiliations:** 1 Department of Materials Science and Engineering, National Tsing Hua University, Hsinchu 30013, Taiwan; 2 High Entropy Materials Center, National Tsing Hua University, Hsinchu 30013, Taiwan

**Keywords:** high-entropy polymers, polymer blends, photoluminescence, photonic materials, conjugated polymers, molecular dispersion

## Abstract

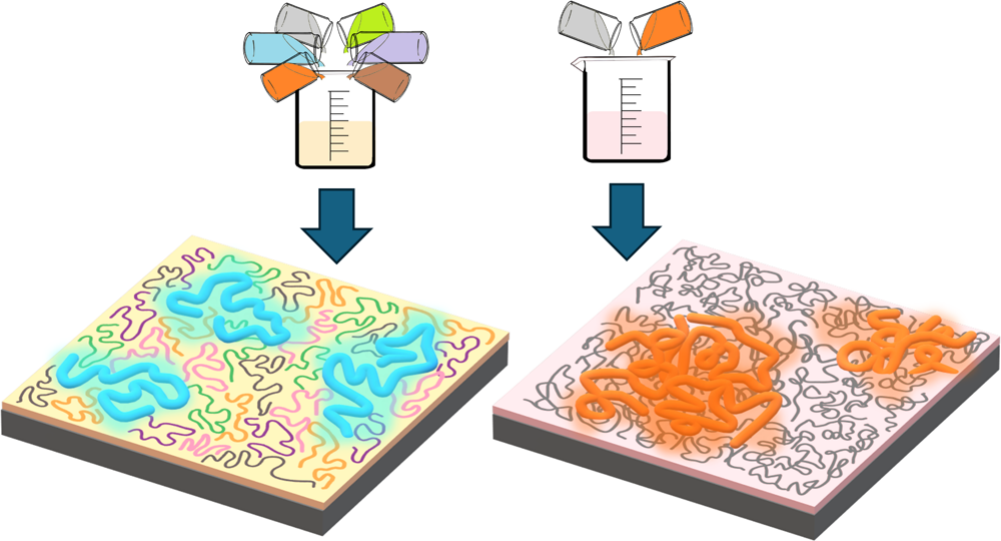

Blending multiple polymers together to form the so-called “high-entropy polymers (HEPs)” can generate the effects of molecular dispersion in addition to suppressing polymer phase separation. We embedded a semiconducting polymer (conjugated polymers, CPs) in an optically inert matrix composed of *n* polymer species and found that a molecule-level dispersion is attained in HEPs defined as *n* ≥ 5. In the regime of dilute CP concentrations, the photonic properties vary widely in the *n* = 1 matrices owing to diverse solubility parameters, but the distribution narrows with *n*, and the CP starts to exhibit behaviors of molecule-level dispersion at *n* ≥ 5, where the matrix polymers compete with each other to exert direct influences on the embedded CP. Specifically, for MEH-PPV, increasing *n* reduces the fluorescence redshift and spectral width from diminishing aggregation. For the rigid PFO molecules, increasing *n* creates a dilution effect facilitating formation of the low-energy planar β-phase. For the flexible regioregular P3HT-rr, HEPs offer well-dispersed amorphous chains highly susceptible to chain environments, thus influencing η_R_’s in the quasi-fixed amorphous–crystalline energy transfer landscape. The HEP effects continue for greater CP concentrations, consistent with the matrix dispersing behaviors in the dilute regime. This work demonstrates a molecular-level dispersion by HEPs, offering a method of molecular tailoring for polymer research and applications via simple mixing.

Over the past two decades, high-entropy alloys have transformed the design and production of metallic alloys, leading to numerous breakthroughs and applications.^1−3^ This concept has recently been applied to polymeric materials, where blending multiple polymer species to form high-entropy polymers (HEPs) does significantly suppress polymer phase separation, a persistent challenge for developing the binary or tertiary polymer blends.^4^ The high-entropy polymers (HEPs) are defined as multicomponent polymer blends with five or more distinct polymer species inspired by the concept of high-entropy alloys. Like high-entropy alloys, generally no single component should dominate the compositions of the HEPs. Since mixing different species together is a simple and cost-effective method for creating new materials, the emergence of HEPs offers an attractive method for developing polymer materials. This study explores the photonic behaviors of light-emitting conjugated polymers (CPs) embedded in HEP thin films. It focuses on how CP molecules interact with the environment to influence their photonic performances, in an attempt to investigate the molecular state on finer lenght scales.

Semiconducting CPs, finding wide applications relating to display, lighting, photodetectors, and microelectronics,^5−7^ are highly susceptible to the states of molecular conformations and aggregation that may substantially impact the energy transfer pathways to dictate the photonic behaviors. For example, segmental stresses arising from nonequilibrated molecular packaging strongly influence the emission wavelength (λ_max_) and the self-trapping propensity; the latter can sabotage the emissive quantum efficiencies (η_R_).^8−16^ Therefore, photonic behaviors are very sensitive to the dispersion state of CP when it is embedded in a matrix. Hence, it would be interesting to study the photoluminescence (PL) of the model CPs of varied backbone stiffnesses and intermolecular properties when embedded in polymer blends of various numbers of components (*n*’s) to scrutinize molecular dispersion as affected by HEPs.

For this purpose, we choose three conjugated polymers of various backbone stiffnesses and photonic properties: the P3HT-rr (regioregular poly(3-hexylthiophene-2,5-diyl)), MEH-PPV (poly(2-methoxy-5-(2-ethylhexyloxy)-1,4-phenylenevinylene)), and PFO (poly(9,9-di-*n*-octylfluorenyl-2,7-diyl)), in the order of increasing backbone rigidity. The CP is then embedded in optically inert polymer matrices composed of varied species from a polymer set of diverse glass transition temperatures (*T*_g_), segmental hydrophobicity, and solubility parameters: polystyrene (PS), poly(methyl methacrylate) (PMMA), *cis*-polyisoprene (PIP), polyvinylpyrrolidone (PVP), poly(vinyl acetate) (PVAc), poly(bisphenol A carbonate) (PC), ethyl cellulose (EC), and poly(2,6-dimethyl-1,4-phenylene oxide) (PPO). Among the CPs, MEH-PPV luminesces in a fashion sensitive to segmental aggregation as the latter can cause redshifted λ_max_ and reduced η_R_ despite a long side group in place designed to mitigate intermolecular π–π attractions.^17−20^ The PFO, a derivative of the blue-emitting polyfluorene family that generally has high η_R_’s,^21−23^ emits different λ_max_’s according to the intermonomer torsion angle ϕ: random ϕ (the α-phase), 140° < ϕ< 160° (the γ-phase), and 160° < ϕ < 180° (the β-phase), with the β-phase emitting the reddest capable to dominate the emission via Forster energy transfer once it exceeds 7% in coexistent states.^24−28^ The β-phase, normally favored due to its relatively greater η_R_, is energetically the lowest, formed preferentially when the polymer chains are plasticized or isolated in diluted states.^24,29−35^ The P3HT-rr is regioregular, capable of interdigitated edge-on crystalline ordering via on-plane π stacking.^36,37^ The π-stacked aggregates lead to extended conjugations that can serve as the “red chains” to funnel photoexcited energies from the “blue chains” residing in the amorphous regions.^38−42^

As shown in the following, HEP blending not only suppresses phase separation of the component polymers but also finely disperses the CP molecules in the matrix, allowing the photonic properties to be tailored by the matrix species, which, at the same time, signifies further the molecular dispersion of the component polymers.

## Results and Discussion

The photonic behaviors of HEPs were examined mainly by blending 1 wt % of a CP (MEH-PPV, PFO, or P3HT-rr) in the optically inert polymer blends to examine the molecular interactions. The CP concentration was ultimately increased to 50 wt % for MEH-PPV to further explore the stability of molecular dispersion. Clearly, as *n* increases, phase separation of the polymers is increasingly suppressed. Although minor demixing of a length scale ∼300–500 nm continues (Figure S1, Supporting Information A), segmental dispersion down to molecular scales is attained in HEPs for both the embedded CP and the matrix polymers, as shown by the photonic behaviors. It implies a molecular dispersion length scale estimated of ∼20 nm via HEPs, much smaller than that implied by the morphology demixing, rendering the photonic behaviors not closely relevant to the morphologies (Figure S2, Supporting Information A).

### PL of Diluted Conjugated Polymers in the Blends

The three CPs of diverse backbone and interchain properties (MEH-PPV, PFO, and P3HT-rr) exhibit distinct dispersion effects unique to each characteristic photonic behavior. The individual behaviors are explored and discussed in the following.

#### MEH-PPV

**Figure 1:**
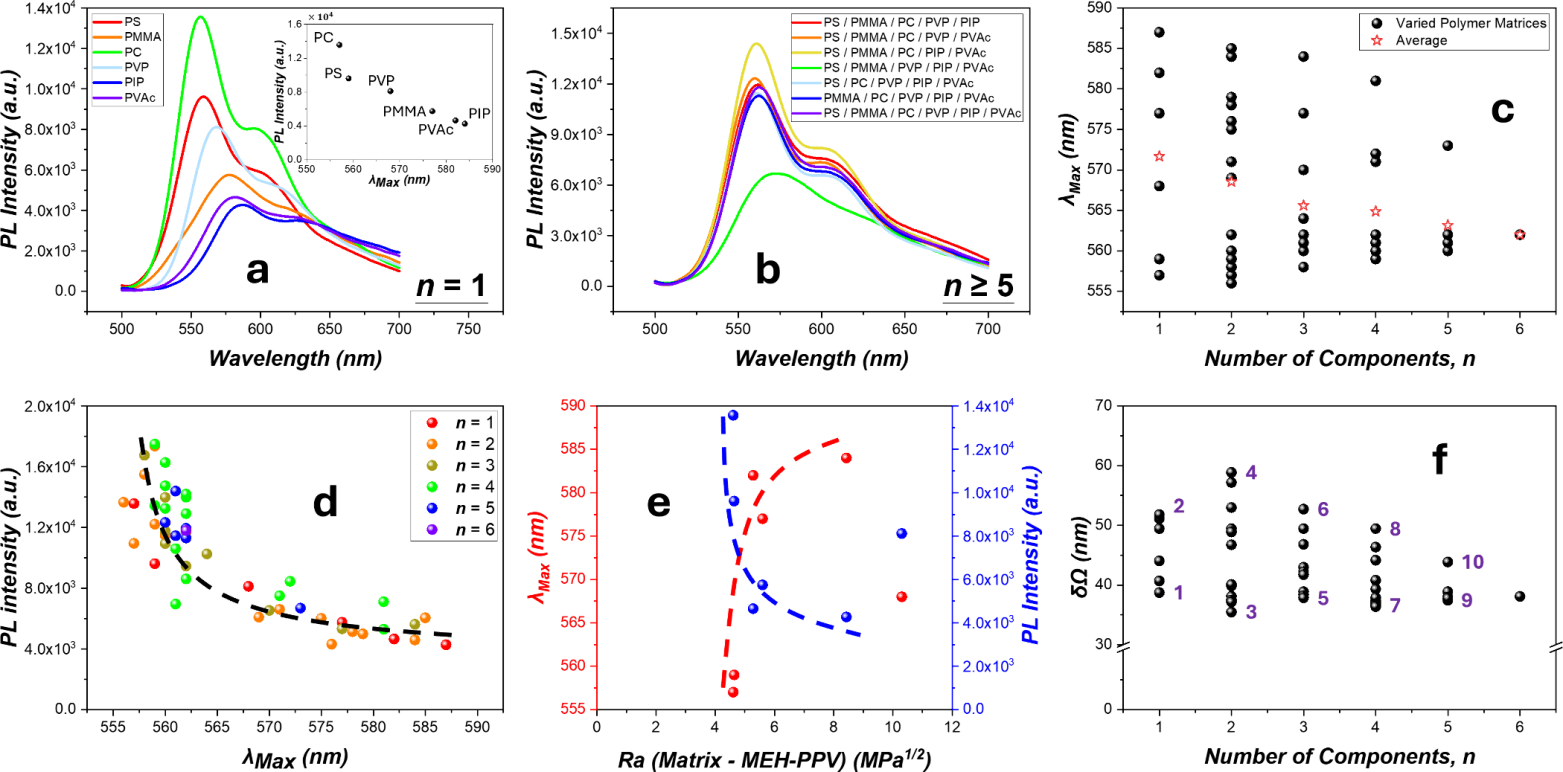
PL spectra of 1 wt % MEH-PPV in the various matrices: (a,b) PL spectra in the *n* = 1 (a) and *n* ≥ 5 (b) polymer matrices. The PL spectra of other *n*’s are shown in Figure S3, Supporting Information B. (c) λ_max_ vs *n* (the polymer compositions of each film can be seen in Figure 2). (d) PL intensity vsλ_max_ for various *n*’s. (e) λ_max_ and PL intensity vs *R*_a_. (f) Breadths δΩ of the 0–0 peaks vs *n*’s. (1: PC, 2: PVAc, 3: PC/PIP, 4: PVP/PIP, 5: PC/PIP/PVAc, 6: PMMA/PIP/PVAc, 7: PC/PVP/PIP/PVAc, 8: PS/PMMA/PVP/PIP and PS/PMMA/PIP/PVAc, 9: PS/PMMA/PC/PIP/PVAc, and 10: PS/PMMA/PVP/PIP/PVAc).

As *n* increases, the embedded CP molecules exhibit significantly reduced redshifts, during which the matrix polymers may influence directly the PL behaviors. At *n* = 1, the PL spectra exhibit distinct emission characteristics with varied degrees of molecular aggregation depending on the matrix polymer species (Figure 1a). In PC or PS, the quasi-single molecules' PL behavior of λ_max_ at ∼557 and 559 nm was observed (single-molecule MEH-PPV emission at λ_max_ = 555 nm),^43^ indicating minimal aggregation in the blends. In contrast, in PIP, PVAc, or PMMA, considerable redshifts were observed (λ_max_ = 587, 582, and 577 nm), which implicate substantial molecular aggregation. The redshifts cause pronounced PL reductions, reflecting aggregation-caused quenching (ACQ) for MEH-PPV optical properties (Figure 1a, inset).

The compatibility between the matrix polymer and CP significantly influences the π aggregation. With PVP being the only exception, λ_max_ increases rapidly with *R*_a_ (the Hansen solubility limit, Table S1, Supporting Information C), as shown in Figure 1e, revealing stronger propensity of intersegmental aggregation for poorer matrix-CP affinities. The observation is consistent with the notion that molecular aggregates arise when the matrix molecules start to pervade and mix with the CP molecules near the end of the solvent evaporation during spin coating. The anomalous deviation by PVP (showing smaller λ_max_ and larger PL intensity than expected on *R*_a_) is tentatively attributed to sizeable separations between the hydrophilic short PVP chains (*M*_PVP_ = 40 kg/mol) and the hydrophobic CP segments during cosolvent-mediated convergence at the final stage of film formation.

For films of *n* > 1, the PL spectra vary remarkably with the matrix polymer composition and *n* (Figure 1b and Figure S3, Supporting Information B), with λ_max_ distribution shrinking with *n* (Figure 1c). The narrowing of the λ_max_ distribution is drastic when *n* increases from 4 to ≥5. It indicates that not only decreased MEH-PPV aggregation but also a convergence into similar emissive structures has occurred as the matrix evolves into the HEP regime.

Moreover, the PL spectral breadth δΩ that reflects the distribution of conjugation lengths exhibits a steep narrowing of its distribution for *n* > 4, after a brief increase at *n* = 2 (Figure 1f). The breadth δΩ is the full width at half-maximum (fwhm) of the 0–0 emission band (Figure S5, Supporting Information D). The fact that δΩ at *n* = 6 is near the minimum for all surveyed samples reveals the uniform emissive states of MEH-PPV in the HEP regime, ruling out the possibility of PL superposition from various phases. Furthermore, the loose correlation between δΩ and λ_max_ (Figure S4c, Supporting Information B), as well as the highest η_R_’s of 17.13% in HEP matrices among all samples (Table S3, Supporting Information E), is attributed to the reduced molecular aggregation.

Like the *n* = 1 samples, the PL intensities correlate with λ_max_ in an exponential decay fashion (Figure 1d) for films of various *n*'s, revealing the pronounced ACQ effect on MEH-PPV optical behaviors. On the other hand, the quantum efficiency η_R_ does not exhibit any good correlation with radiative lifetimes τ_R_'s (Figure S4b, Supporting Information B); rather, it correlates loosely in a negative fashion with the nonradiative lifetimes τ_NR_’s (Figure S 4d, Supporting Information B), indicating that nonradiative events strongly impede the emission processes.^9,44^

**Figure 2:**
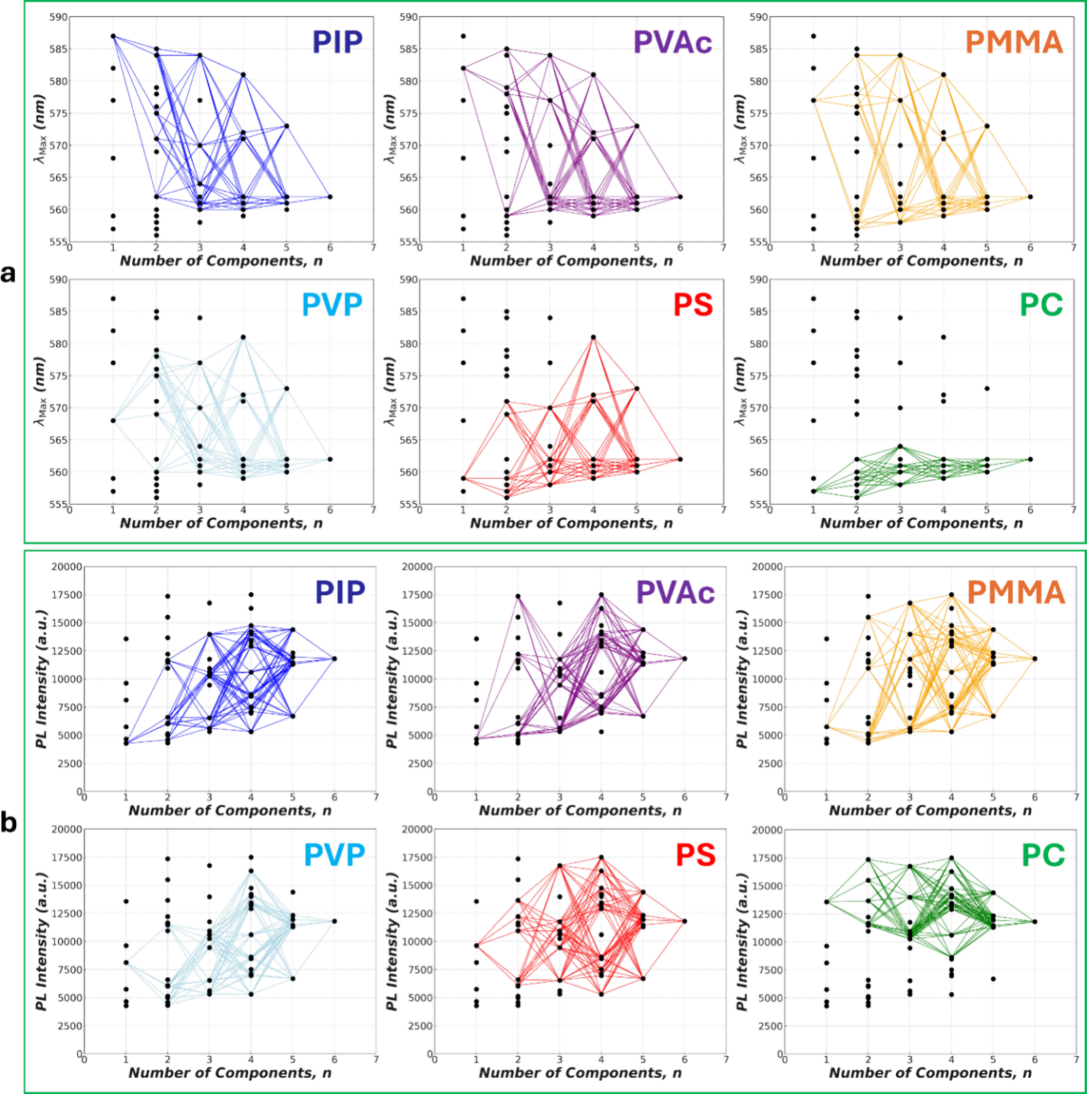
Detailed tracing of the λ_max_’s (a) and the PL intensities of the 0–0 transition (b) of the 1.0 wt % MEH-PPV for each polymer component in the various matrices of varying *n*.

We further surveyed λ_max_ across all *n*'s for each matrix species (Figure 2a). We found that the polymer blends containing PC always exhibit the lowest λ_max_’s, indicating that PC facilitates the reduction of MEH-PPV aggregates even when the PC fraction dwindles in the HEP regime. This implies effective dispersion of the matrix species itself in addition to CP dispersion in HEPs. Conversely, the films containing PIP always manifest the highest λ_max_'s unless they also contain PC, indicating PIP behaving as a CP aggregation promoter while PC can override the effect for all *n*’s. Along the same line, those exhibiting intermediate λ_max_’s at *n* = 1 also demonstrate intermediate λ_max_’s in their blends unless they are blended with PC or PIP.

Similarly, we found that all films that contain PC always deliver among the highest PL intensities, while those containing PIP always deliver the lowest unless blended with PC (Figure 2b). The polymers of intermediate PL intensities at *n* = 1 also manifest the intermediate PL intensities in the blended films unless they are blended with either PIP or PC. This reiterates molecular-scale dispersion of each matrix species in the HEP regime, allowing specific segmental interactions to influence the photonic behaviors of MEH-PPV.

#### PFO

**Figure 3:**
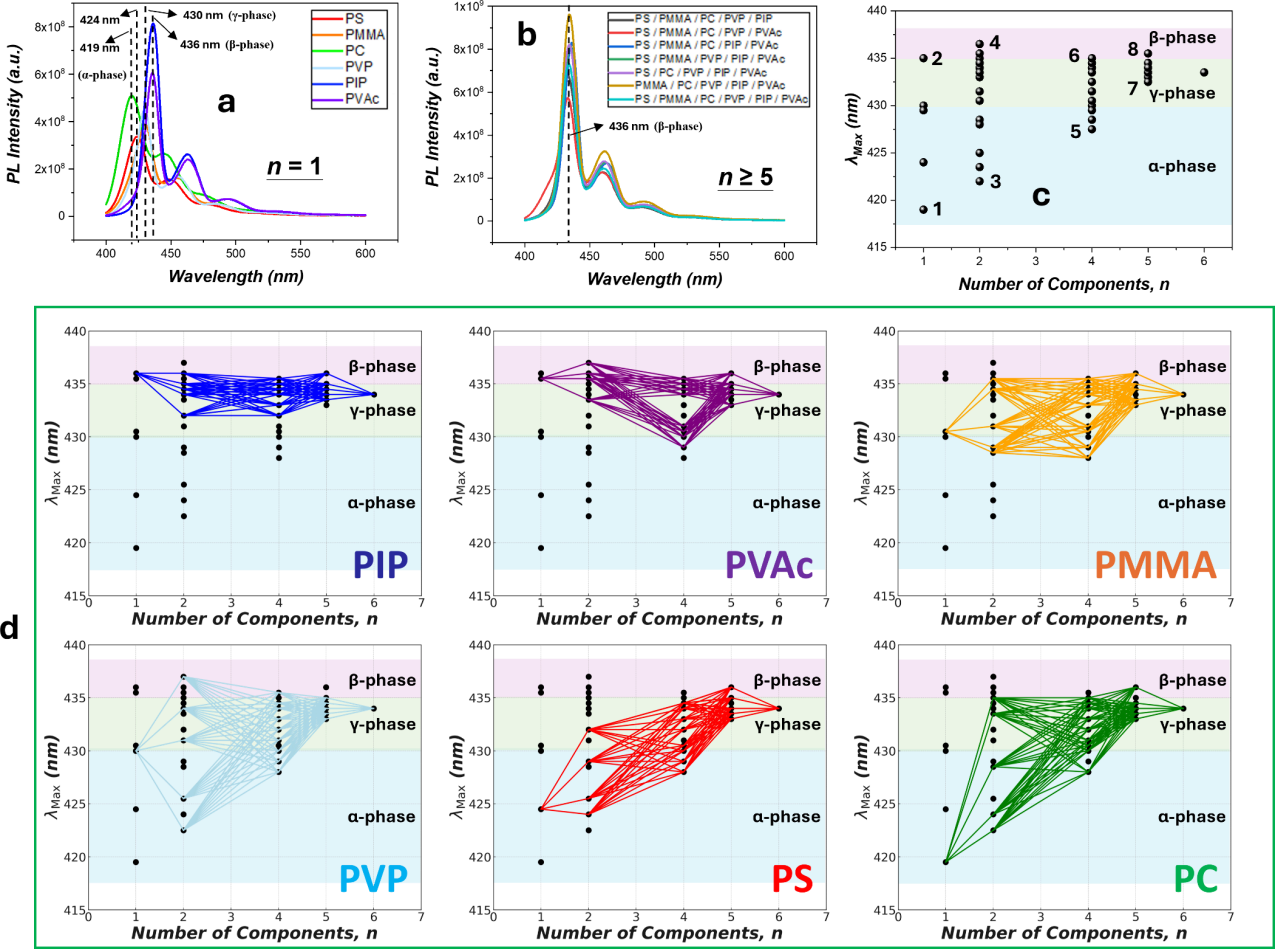
(a) PL spectra of 1 wt % PFO in the various *n* = 1 matrices, showing distinct phase behaviors of the amorphous α-phase, γ-phase, and planar β-phase. (b) PL spectra of 1 wt % PFO in HEPs (comprising *n* = 5 and 6), exhibiting the formation of the β-phase. (c) λ_max_’s of 1 wt % PFO in the various polymer matrices. 1: PC, 2: PIP, 3: PC/PVP, 4: PVP/PVAc, 5: PS/PMMA/PC/PVP, 6: PMMA/PVP/PIP/PVAc, 7: PS/PMMA/PC/PVP/PVAc, and 8: PS/PMMA/PC/PIP/PVAc. (d) Tracing on λ_max_’s vs *n* for each polymer component.

The PFO molecules, in contrast, interact differently with the HEP matrices to affect the photonic behaviors owing to their more rigid backbones and shorter conjugation lengths. As shown in Figure 3a, at *n* = 1, the PFO exhibits λ_max_ = 417 nm of the 0–0 band in the PC matrix, revealing a metastable amorphous α-phase. The emission redshifts to λ_max_= 424 nm in PS, indicating an incipient degree of order of the largely amorphous PFO chains. The chain order is further enhanced in PMMA and PVP, with the PFO adopting the γ-phase emitting λ_max_ = 430 nm. Finally, the chain order grows in PIP and PVAc, exhibiting λ_max_ = 436 nm to signify formation of the planar β-phase in these matrices.

The emission phases at *n* = 1 appear to reflect the extent of PFO segmental relaxation during interactions with the matrix polymer during spin coating. In that, we believe that the low *T*_g_ matrices, such as PIP (*T*_g_= −67 °C) and PVAc (*T*_g_= 42 °C), provide the environments for PFO chains to relax to the low-energy planar β-phase (λ_max_ = 436 nm), an effect akin to that by high-boiling-point solvent residuals (such as isodurene and cyclopentanone) in PFO films.^45^ Conversely and consistently, the metastable α-phase (λ_max_ = 417 nm) prevails in the highest *T*_g_ matrix of PC (*T*_g_= 147 °C). In matrices of intermediate *T*_g_’s between 100 and 120 °C (PS, PMMA, and PVP), λ_max_ emerges between 424 and 430 nm, a spectral range between the emissions of the α- and β-phases.

As *n* increases beyond *n* = 1, the PL spectrum evolves toward the β-phase (Figure 3b,c and Figure S8, Supporting Information F), with λ_max_'s narrowing their distribution that finally converge to well above 430 nm at large *n*’s, testifying the prevalence of the planar phase (Figure 3c). The dominance of the β-phase in the HEP matrices (Figure 3b) indicates an HEP environment promoting chain relaxation to the low-energy planar order, for which we assert that the molecular-level dispersion conferred by HEPs renders the CP to interact with the low-*T*_g_ matrix polymers, producing an effect similar to that at *n* = 1 even with much less fractions of the matrix polymer at higher *n*’s. The detailed tracings in Figure 3d consistently illustrate the persistence of such CP-matrix polymer interactions that influence PFO chain relaxation up to the HEP regime. Since the β-phase is commonly associated with respectable quantum yields η_R_'s (Figure 3a),^24−35^ the HEP strategy may help enhance PFO photonic performance, although other molecular interactions may also simultaneously influence η_R_.

#### P3HT-rr

**Figure 4:**
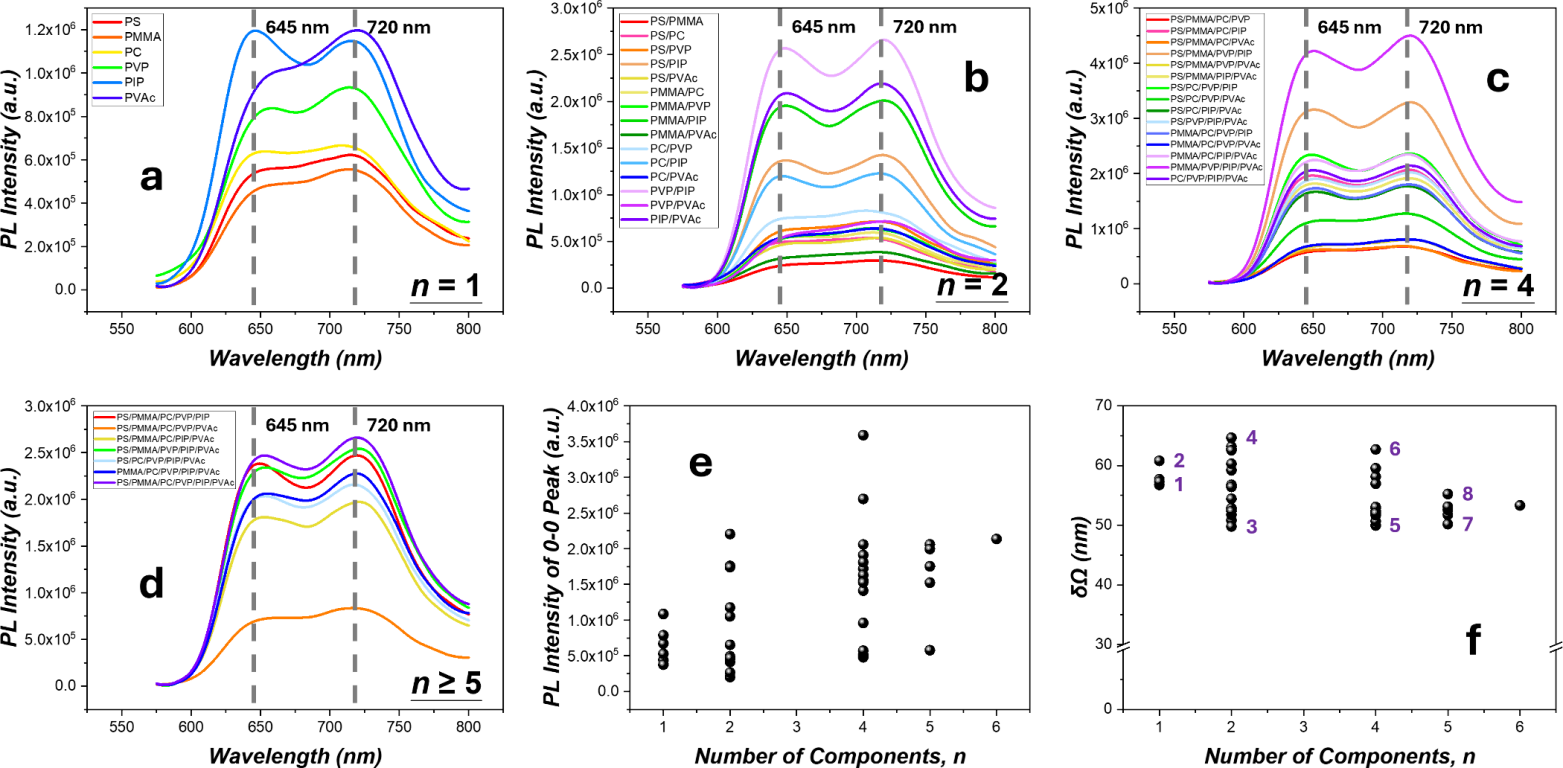
PL of the 1.0 wt % P3HT-rr in the various polymer blends: (a–d) PL spectra in *n* = 1 (a), *n* = 2 (b), *n* = 4 (c), and *n* ≥ 5 (d) matrices; (e) PL intensity in the various matrices vs *n*. The PL intensity across all *n*'s for every specific matrix species can be seen in Figure S9, Supporting Information G. (f) Breadth of the 0–0 peak δΩ in the different matrices vs *n*. 1: PIP, 2: PVP, 3: PVP/PIP, 4: PS/PVAc, 5: PS/PMMA/PVP/PIP, 6: PS/PMMA/PC/PVAc, 7: PS/PMMA/PC/PVP/PIP, and 8: PS/PMMA/PC/PVP/PVAc.

P3HT-rr, owing to its soft backbones and strong intersegmental ordering, presents a window for investigating how polymer crystallinity influences photonic behaviors in HEP environments. In contrast to the behaviors of MEH-PPV and PFO, the PL spectra in the various *n* = 1 matrices reveal a conspicuously constant spectral shape with little peak shifts (Figure 4a). It suggests the existence of a stable emission structure. Moreover, P3HT-rr molecules in solid matrices form a morphology composed of isolated amorphous chain segments interspersed within aggregated phases of varied orders. Since ordered chains offer longer conjugation lengths,^41^ they may serve as the “red chains” to funnel photoexcited energies, including that absorbed by the amorphous “blue chains”, into photon emissions. This stable energy transfer (Forster resonance energy transfer, FRET) effectively defines a constant emission structure that could have an effect on the PL spectrum. Notably, the light-emitting crystalline structures in this context are highly “preserved” to secure a quasi-constant emission spectral shape in the various matrices.

The PL intensities, however, vary enormously in the different matrix polymers at *n* = 1 (Figure 4a). The intensity variation seems to arise from changes in the amorphous environments that affect the η_R_. We observed that the “softer” chain environments generally confer more efficient emissions, in that the rubbery PIP (*T*_g_= −67 °C) gives the most efficient emissions followed by the low-*T*_g_ polymer of PVAc (*T*_g_ = 30–45 °C). Conversely, the glassy polymers of PS and PMMA (PS: *T*_g_ = 100 °C, PMMA: *T*_g_ = 105 °C) afford the chain environments giving the lowest PL emissions. Consistently, lying in between are PVP and PC matrices, glassy but of lower molecular weights (PVP: *T*_g_ = 120 °C, 40 kg/mol, PC: *T*_g_ = 147 °C, 45 kg/mol), following the trend that “plasticizing” environments result in higher PL emissions for the amorphous P3HT-rr chains.

Furthermore, the generally low η_R_’s of P3HT-rr (∼2% for pristine P3HT-rr films) indicate that the excited states of P3HT-rr predominantly relax nonradiatively with rampant self-trapping.^9,16^ Since the strain energies causing self-trapping are mitigated by increased segmental flexibility,^9,16^ the softer chain environments would allow the amorphous chains to exhibit more effective energy transfer to the emissive crystalline aggregates. In addition, enhanced energy transfer may have contributed to the higher 0–0 band (645 nm) relative to the 0–1 band (720 nm) in PIP (Figure 4a).^41,46−48^ For P3HT, varied distributions of the J- and H-aggregates as proposed in the literature may also play a role in the emission behaviors, nevertheless.^47^

For samples of *n* > 1, the same spectral shape persists, with emission peaks at 645 and 720 nm corresponding, respectively, to the 0–0 and 0–1 bands, identical to those of the *n* = 1 samples (Figure 4b–d). We also found that the molecular factors influencing PL efficiency at *n* = 1 continue to operate for *n* > 1, in that the matrices containing rubbery PIP chains exhibit the highest PL intensities among the blends of the same *n* for all *n*'s (Figure 4b–d and Figure S9, Supporting Information G). In addition, the PL intensities generally increase with *n*, except for an anomaly at *n* = 4, and reach a relatively high value at *n* = 6 (Figure 4e). The increasing trend of PL with *n*, recalling that η_R_ of P3HT-rr increases with molecular dilution (from η_R_ ∼ 2% in the pristine state to η_R_ ∼ 21% as diluted at 0.1 wt % in PS),^16^ implies a dilution effect derived from effective molecular dispersion as *n* increases. The moderate anomalous downturn beyond *n* = 4, on the other hand, seems to indicate the existence of a threshold fraction of PIP for effective η_R_ enhancement, below which (∼25 wt %) the enhancement may start to decline. Remarkably, that a small fraction of the softening polymer at *n* = 4 (∼25 wt % PIP) can generate more than 3.5-fold PL enhancements relative to that in the *n* = 1 matrix (100% PIP) indicates the prominent molecular dispersion in HEPs.

In addition, a trend of narrowing δΩ distribution with *n* was observed (Figure 4f), which was akin to that of MEH-PPV (Figure 1f). The narrowing underscores the uniformity of emissive states as dictated by the crystalline aggregates. It also indicates the relatively minor impact due to changes of the polymer matrix to the emissive crystalline morphology and consequently the δΩ, in contrast to that in MEH-PPV where the emissive noncrystalline aggregates are highly sensitive to matrix interactions and hence give rise to broader δΩ distribution (22 nm for MEH-PPV vs 15 nm for P3HT-rr at *n* = 2).

### PL of Concentrated Conjugated Polymers in the Blends

The photonic behaviors of greater CP concentrations (*c*’s) up to *c* = 50 wt % were further explored for MEH-PPV, where two *n* = 5 high-entropy polymers (HEP-1 and HEP-2; HEP-1: equal-parted PS, PMMA, PC, PVP, and PIP; HEP-2: equal-parted PS, PMMA, PC, PVP, and PVAc) were prepared.

**Figure 5:**
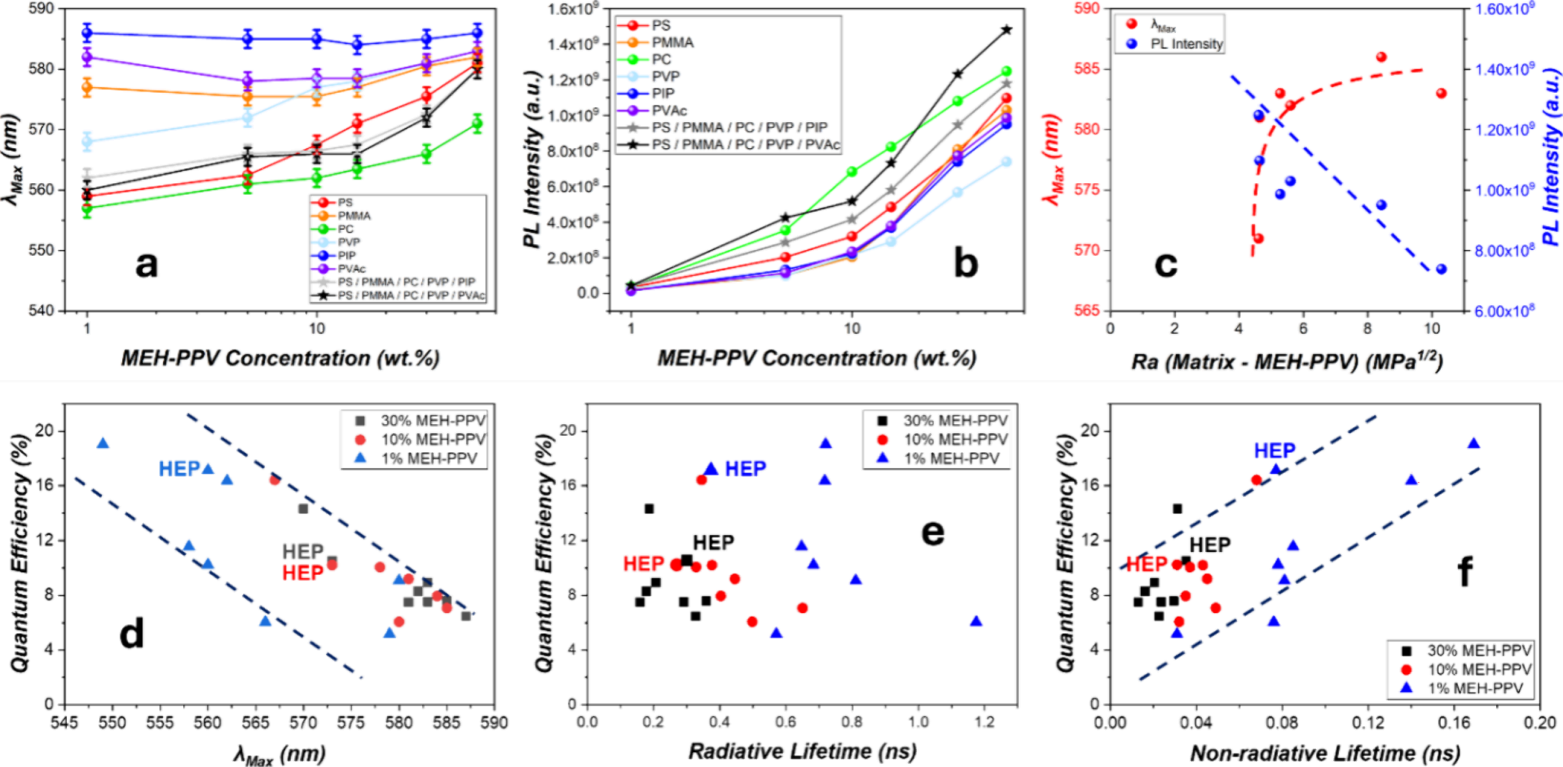
(a,b) λ_max_ (a) and PL intensity (b) vs MEH-PPV concentration in the various *n* = 1 matrices as well as the two *n* = 5 HEP matrices (HEP-1: equal-parted PS, PMMA, PC, PVP, and PIP; HEP-2: equal-parted PS, PMMA, PC, PVP, and PVAc). (c) Correlations between the emission properties (PL intensity and λ_max_) and *R*_a_ for the 50 wt % MEH-PPV in the *n* = 1 matrices. (d–f) η_R_ vs λ_max_ (d), radiative lifetime τ_R_ (e), and nonradiative lifetime τ_NR_ (f) for MEH-PPV of various concentrations (1, 10, and 30 wt %) in the *n* = 1 and *n* = 7 matrices, with *n* = 7 data marked.

The MEH-PPV PL spectra in these systems exhibit an approximately constant spectral shape vs *c,* featuring a prominent 0–0 band and a lesser 0–1 band (Figure S10, Supporting Information H). The λ_max_’s, however, vary in different *n* = 1 matrices and undergo redshifts as *c* increases (Figure 5a). In the PIP matrix, λ_max_ stays at ∼590 nm in the full *c* range from 1 to 50 wt %. Since the pristine MEH-PPV also emits at 590 nm,^9^ it seems to indicate that MEH-PPV always forms certain characteristic molecular aggregates in PIP independent of *c* to dominate the emissions. In contrast, MEH-PPV disperses to various degrees in other polymers, e.g., in the PC matrix, the emissions are located at λ_max_ = 557 nm for 1 wt %, very close to MEH-PPV single-molecule emissions (∼555 nm), then increase slowly to 571 nm at 50 wt %, signifying the progressive CP aggregation as *c* increases. With λ_max_ being the reddest in PIP, always staying at ∼590 nm, while others undergo redshifting with *c*, the distribution of λ_max_ thus narrows as *c* increases (Figure 5a). In the concentrated CP regime, λ_max_ correlates well with the Hansen solubility distance *R*_a_ (Figure 5c), similar to that in the 1 wt % diluted systems (Figure 1e), signifying lesser aggregation for smaller *R*_a_’s, and vice versa, for CP mixing with the matrix polymer.

In the high-entropy polymers (HEP-1 and HEP-2), λ_max_’s persistently exhibit the lowest among films of the same CP concentration in the entire explored *c* range. In the *c* range from 1 to 15 wt %, dubbed the HEP regime where no polymer species overwhelm the others, the λ_max_’s remain almost constant with *c*, ranging in a small span of ∼562–567 nm (Figure 5a). As *c* increases, the λ_max_’s then increase slowly to 580 nm at *c* = 50 wt %. The emission peaks λ_max_’s of the HEP-1 and HEP-2 are nearly indistinguishable, indicating that swapping PIP with PVAc produces insignificant changes in terms of CP aggregation up to *c* = 50 wt %, testifying the robust suppression of CP phase separation in the HEP systems. The CP dispersion persistent into the high *c*’s agrees excellently with the observed molecular dispersion of the component polymers in the HEP matrices and by which the component polymer can directly influence the photonic behavior of the embedded CP molecules.

The PL intensity of any of these concentrated samples increases with *c* (Figure 5b), indicating that the rates of aggregation-caused quenching (ACQ) vs *c* for MEH-PPV are slower than that on increasing emissive species. Nevertheless, at *n* = 1, the PL intensity is among the lowest for CP in PIP and at the same time the highest in PC for the whole explored *c* range, consistent with the correlation of λ_max_ with *R*_a_ (Figure 5c), like that in the diluted systems (*c* = 1 wt % vs *n*, Figure 2b), which clearly demonstrates the strong effect of polymer miscibility on PL intensity under the influence of ACQ. In the high-entropy polymers, the CP emits among the highest, believably owing to the more dispersed states of the CP molecules. In the concentrated films of 30 and 50 wt %, the HEP-2 emits moderately stronger than HEP-1, a behavior to be explored further but is tentatively attributed to lower residual stresses in the rubber-containing HEP-1 films.^13−16^

### Quantum Efficiencies, Luminescence Lifetimes, and Some General Discussions

We further analyzed quantum efficiencies η_R_’s and luminescence lifetimes τ’s of MEH-PPV in the *c* range up to 30 wt % at *n* = 1 and *n* = 7, examining the HEP effects. We found that η_R_ decreases continuously with λ_max_ from ∼20 to ∼5% as the latter increases from ∼550 to 587 nm (Figure 5d), which can be interpreted as due to aggregation-caused quenching (ACQ). Furthermore, we found that greater *c*'s are generally associated with shorter radiative lifetimes τ_R_ (Figures S6 and S7 and Tables S2–S5, Supporting Information E), and η_R_ does not correlate well with τ_R_'s (Figure 5e), although generally shorter τ_R_'s are believably correspond to greater propensities for radiative decay. Instead, η_R_ seems to increase with decreasing nonradiative lifetime τ_NR_ (Figure 5f), suggesting that the nonradiative pathways are competing strongly with the radiative processes in the CPs explored here.

In light of this observation, we also noticed that η_R_’s of MEH-PPV in HEP matrices are among the highest, and there is a positive correlation between η_R_ and τ_ΝR_ across all films, including HEPs. This suggests that better molecular dispersion via HEPs suppresses nonradiative pathways. Specifically, molecular dispersion attained by HEP mixing facilitates β-phase formation for PFO and allows segmental plasticization for amorphous P3HT-rr chains, both producing the effects of improved η_R_.

Molecular dispersion via HEPs, in essence, is derived from diminishing encounter probabilities between like-polymers during processing at large *n*’s. This behavior is independent of the choice of component polymers and thus can apply to other multiple polymer systems in general. Although good dispersion conceptually would lead to the average over that of the individual components, if a strong dependence arises for a specific photonic perofrmance on molecular isolation, molecular dispersion via HEPs would engender the best or among the best performance results. In addition, when prominent interactions prevail conferred by some specific component polymers, the robust interactions would sway the averaged results. The phase behaviors of PFO and P3HT-rr as affected by PIP plasticization belong to the latter, while λ_max_ and η_R_ of MEH-PPV the former. The averaging effect would also result in decreased spreads of photonic behaviors in general, unless phase separation or new phases cause an increase in the spread at small *n*’s before the final converging at large *n*’s when molecular dispersion via HEPs dominates.

Note that this work adopted a simplified “equal-parted approach” for analyzing the HEP blending, which only serves as a stepping stone for further in-depth explorations. For example, as illustrated by the “tracing” of each photonic attribute vs *n* for specific component polymers (Figures 2 and 3b and Figure S9, Supporting Information G), we can identify the favorable or unfavorable matrix polymers and then go on to adjust the compositions or modify the choices of component polymers for optimized performances.

Obviously, owing to the capability of precision tailoring via molecular tuning, HEPs are expected to be useful in many applications where polymers are already being used, in addition to the areas that call for new functions, potentially, e.g., optoelectronic devices, barrier materials, biomedical materials, stimulus-responsive materials, polymer compatibilizers, and recycling without classification, among others. However, further research and development endeavors are required to expand these possibilities. Capable of molecular-scale dispersion even for dissimilar polymers, simple blending via HEPs offers a method of molecular tailoring for polymer research and applications.

## Conclusions

By studying the photonic behaviors of conjugated polymers (CPs) embedded in matrices of varied numbers of polymer species (*n*’s), we demonstrate that molecule-level dispersion of polymers can be attained in the high-entropy polymer (HEP) regime defined as *n* ≥ 5. For a dilute concentration of CP (*c* = 1 wt %), the photonic properties vary widely in the *n* = 1 matrices owing to the diverse solubility parameters, but the distribution narrows with *n*, and the CPs start to exhibit behaviors of molecule-level dispersion at *n* ≥ 5, where the matrix polymers compete with each other to exert direct influences on the embedded CP. Specifically, via HEPs, the MEH-PPV exhibits quasi-single-molecule emissions, the PFO demonstrates β-phase fluorescence upon molecular dilution, and the P3HT-rr renders well-dispersed amorphous chains highly susceptible to segmental plasticization. For higher CP concentrations, molecular-level dispersion also arises showing effects of molecular dispersion for *c*’s up to ∼15–20 wt %, consistent with the matrix dispersion behaviors operative in the dilute regime. With CP molecules still finely dispersed in HEPs, the photonic performances are enhanced up to *c* = 50 wt %. Based on the simple principle of diminishing encounter probabilities between like-polymers during processing, the HEP strategy offers a method of molecular tailoring via simple mixing.

## Experimental Section

### Chemicals and Materials

Eight optically inert polymers were selected for constructing the polymer matrices: polystyrene (PS, *M*_w_ = 123,000 g/mol), poly(methyl methacrylate) (PMMA, *M*_w_ = 350,000 g/mol), poly(bisphenol A carbonate) (PC, *M*_w_ = 45,000 g/mol), polyvinylpyrrolidone (PVP, *M*_w_ = 40,000 g/mol), *cis*-polyisoprene, (PIP, *M*_w_ = 35,000 g/mol), poly(vinyl acetate) (PVAc, *M*_w_ = 100,000 g/mol), poly(2,6-dimethyl-1,4-phenylene oxide) (PPO, *M*_w_ = 244,000 g/mol), and ethyl cellulose (EC, 48% ethoxyl), all purchased from Sigma-Aldrich except that PS was bought from Pressure Chemical Co. For the three conjugated polymers (CPs), PFO (poly(9,9-di-*n*-octylfluorenyl-2,7-diyl), *M*_w_ = 74,766 g/mol, PDI = 3.68) was purchased from Ossila Ltd., MEH-PPV (poly(2-methoxy-5-(2-ethylhexyloxy)-1,4-phenylenevinylene), *M*_n_ = 150,000–250,000 g/mol) was bought from Sigma-Aldrich, and P3HT-rr (poly(3-hexylthiophene-2,5-diyl), regioregular, *M*_w_ = 58,000 g/mol) was obtained from Rieke Metals. Chloroform (ACS grade) used as a cosolvent for all these polymers was purchased from Sigma-Aldrich.

### Preparation of Solutions and Films

Each polymer was dissolved separately in chloroform before mixing and casting into thin films. The hygroscopic polymers (PMMA, PPO, and PVP) were dried at 85 °C under vacuum for 24 h to remove moisture before solution preparation. The solution of each matrix polymer was prepared by stirring at 25–30 °C for a day in ambient conditions, while the CP solutions were prepared by stirring at 35 °C for a day under wrapped aluminum foil in a nitrogen-maintained glovebox controlled under 3 ppm for both oxygen and moisture. For the *n* = 1 samples, the solution of the matrix polymer was added to the CP solution at an amount according to the desired concentration and stirred for half a day before spin coating into films. For the *n* > 1 samples, the matrix polymer solutions were first mixed in equal parts before adding the CP solution. Spin coating was carried out at 4000 rpm for 20 s, producing films of 35–45 nm thickness on a glass slide. The film thickness was determined using a scanning probe microscope (Icon, Bruker, the Instrument Center at NTHU). Multiple duplicated samples were prepared and tested to ensure data reproducibility. No new polymer species or chain cross-linking was produced via any chemical reactions from the sample preparation.

### Characterization of the Ultrathin Films

The topography and phase images of the samples were examined also using the scanning probe microscope (Icon, Bruker, the Instrument Center at NTHU). The AFM data were further processed via a fast Fourier transform for quantitative analyses. The photoluminescence (PL) spectra were obtained by using a Horiba FluoroLog-3 (NanoLog-3) PL spectrometer, excited at 380, 480, and 550 nm, respectively, for PFO, MEH-PPV, and P3HT-rr. The quantum efficiencies (η_R_’s) were obtained also by using the PL spectrometer with the equipped integrating sphere accessory, the errors of determination being around ∼3–5%. The fluorescence radiative lifetimes (τ_R_) were measured using a time-correlated single-photon counting setup (TCSPC, the Instrument Center at NTHU), excited at 405 nm and detected at 560 and 437 nm, respectively, for MEH-PPV and PFO, using the PicoHarp software for data fitting. The nonradiative lifetime was then calculated from τ_R_ and η_R_ using the conventional equation.

## Supporting Information Available

Film morphology vs PL spectra, photonic properties of the 1.0 wt % MEH-PPV in the various polymer blends, solution properties of the polymer components, determination of δΩ and fwhm of the 0–0 band, luminescence lifetimes and quantum efficiencies, photonic properties of the 1.0 wt % PFO in the various polymer blends, photonic properties of the 1.0 wt % P3HT-rr in the various polymer blends, and photonic properties of concentrated CP in the polymer matrices (PDF)
