# Supplementary material for: Photonics of High-Entropy Polymers Revealing Molecular Dispersion via Polymer Mixing
Source: ACS Nano. 2024 Nov 16;18(47):32759–68. doi: 10.1021/acsnano.4c10585 (PMC11603877; doi:10.1021/acsnano.4c10585)
Supplement: Supplementary file 1 — nn4c10585_si_001.pdf [file nn4c10585_si_001.pdf]

Supporting Information

for

Photonics of High-Entropy Polymers Revealing Molecular Dispersion via Polymer Mixing

Yu-Jr Huang,[1] Jien-Wei Yeh,[1, 2] Arnold Chang-Mou Yang[1*]

[1] Department of Materials Science and Engineering, National Tsing Hua University, Hsinchu, Taiwan, 30013, ROC

[2] High Entropy Materials Center, National Tsing Hua University, Hsinchu, Taiwan, 30013, ROC

Corresponding author. Email: acyang@mx.nthu.edu.tw

A. Film morphology *vs.* PL spectra:

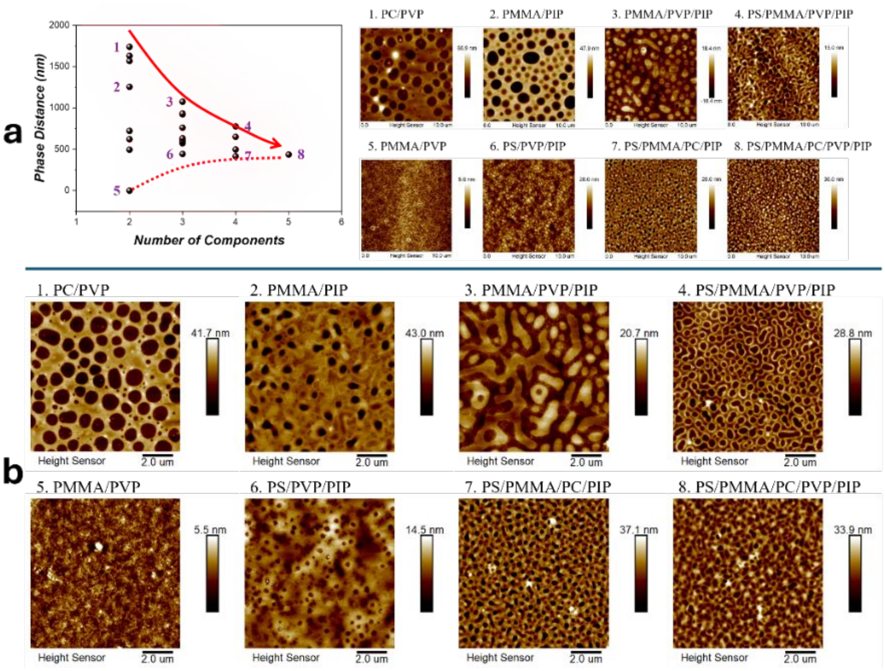

Figure S1. The topography of the polymer blends: (a) without the 1 wt.% MEH-PPV, [4] (b) with the 1 wt.% MEH-PPV. The unusually low phase distance of PMMA/PVP blend, deviating from the overall

trend of increasing ***n***, is an anomaly arising from hydrogen bonding between the carbonyl groups of PMMA and the pyrrolidone groups of PVP that has enhanced the miscibility.[S1]

[S1] Hsu, W.-P. Effect of Tacticity of Poly(Methyl Methacrylate) on the Miscibility with Poly(Vinyl Pyrrolidone). *J. Appl. Polym. Sci.* **2001**, 81, 3190-3197.

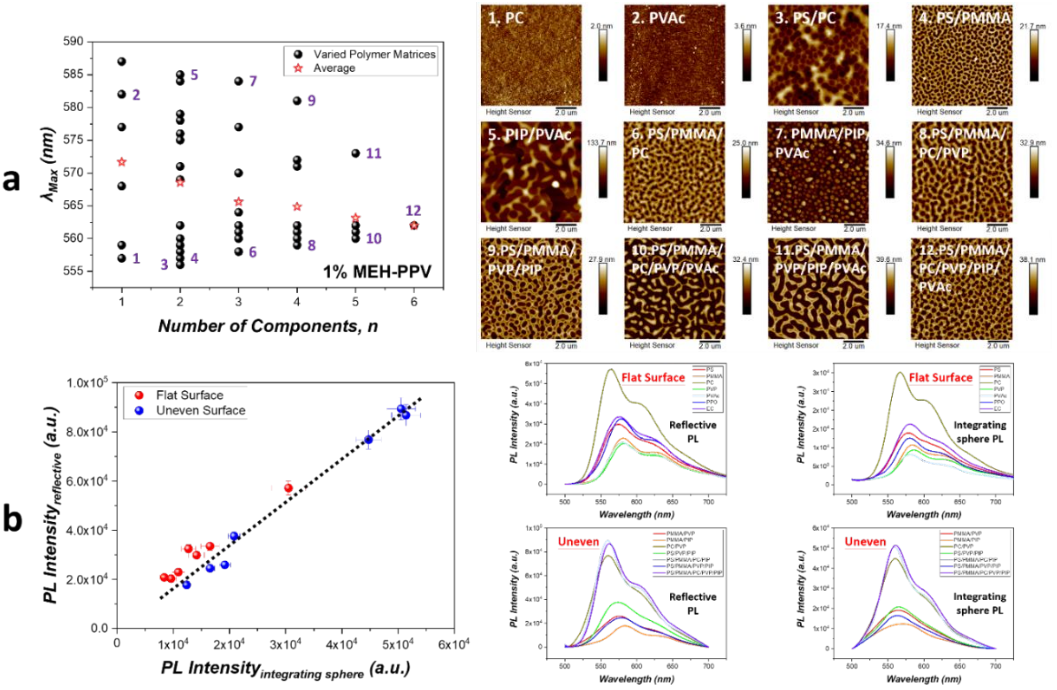

Figure S2. (a) The polymer blends' emission peak λ_{max}'s as a function of ***n*** (the number of polymer components in the blend) and AFM topographic micrographs of selected samples. (b) the PL intensity of a film acquired through standard reflective mode versus the PL intensity of the same film using an integrated sphere for various polymer blend samples. The red dots refer to the ***n*** = 1 films that have smooth surfaces, while the blue dots refer to polymer blends that generally have surface undulation. The comparison indicates that the surface undulations do not affect the measured PL intensities.

The blend films may exhibit uneven exteriors showing surface undulations or patterned microstructures, but these surface features have demonstrated no direct effects on the luminescence of the films. As shown in Fig. S1a, the λ_{max} of the PL spectrum bears no correlations with the AFM topography, and the PL intensities of these rugged films scale identically with that of the smooth films

(***n*** =1) in the comparison between the PL acquired via the common reflection mode and that through the integration sphere method (Fig. S1b). (This indicates that the length scales dominating luminescence are much smaller than the unevenness, and the film roughness produces insignificant scattering to affect the PL intensity.)

B. Photonic Properties of the 1.0 wt.% MEH-PPV in the Various Polymer Blends:

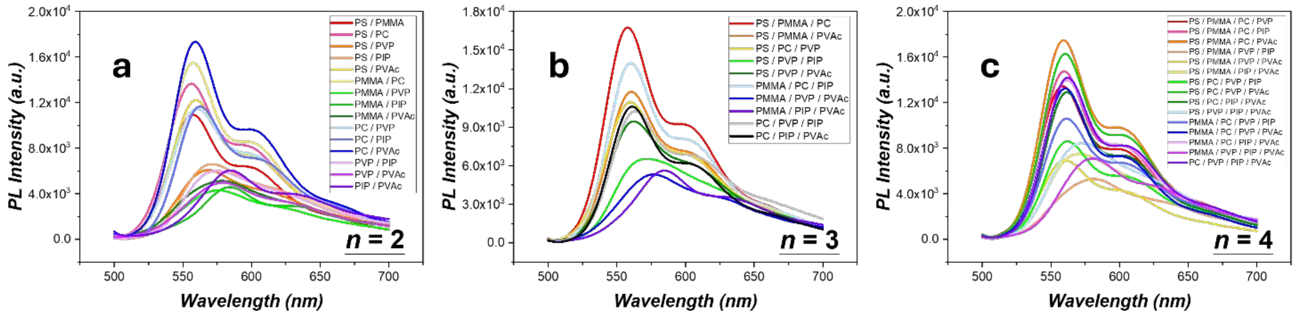

Figure S3. (a-c) The PL spectra of the 1 wt.% MEH-PPV in the ***n*** =2, 3, 4 polymer blends.

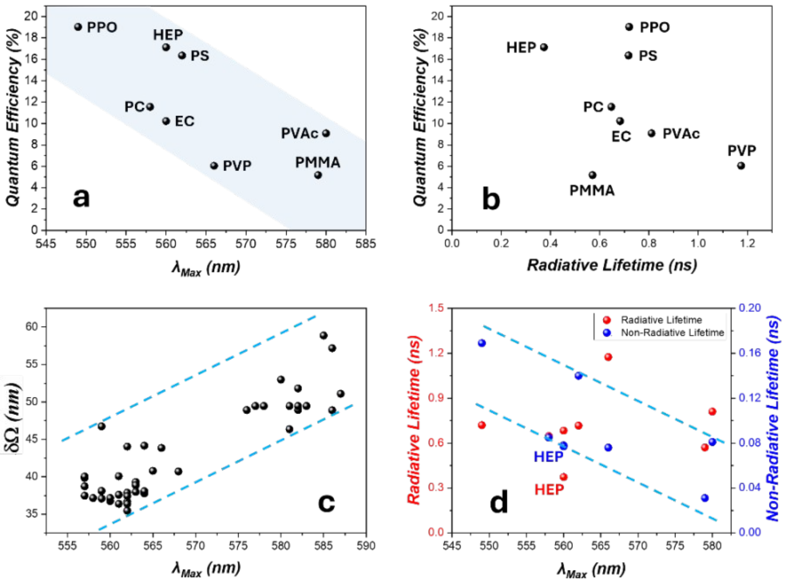

Figure S4. (a) The quantum efficiency η_R *vs.* λ_{max} of the 1.0wt.% MEH-PPV in the ***n*** =1 films. Note that PIP is "flowing" at ***n*** = 1, rendering the determination of η_R in the PIP matrix impossible. (b) Quantum efficiency η_R *vs.* radiative lifetime τ_R of 1 wt.% MEH-PPV in different ***n*** = 1 and the ***n*** = 7

matrices. (c) The spectral breadth $\delta\Omega$ *vs.* λ_{max} of 1 wt.% MEH-PPV in the various matrices. (d) The radiative lifetime τ_R and the non-radiative lifetimes τ_{NR} *vs.* λ_{max} of the 1 wt.% MEH-PPV in different ***n*** = 1 and the ***n*** = 7 matrices. (***n***=7 refers to the blend of PS/PMMA/PC/PVP/PPO/PVAc/EC)

C. Solution Properties of the Polymer Components:

Table S1. The Hansen solubility parameters (δ_d, δ_p, δ_h) and glass transition temperature (T_g) of the polymer components in the blends,[S2-S7] as well as the R_a values respective to MEH-PPV (δ_d=19.06 MPa$^{1/2}$, δ_p=5.38 MPa$^{1/2}$, δ_h=5.44 MPa$^{1/2}$) and PFO (δ_d=18.55 MPa$^{1/2}$, δ_p=2.8 MPa$^{1/2}$, δ_h=4.51 MPa$^{1/2}$).

Polymer	**PS**	**PMMA**	**PC**	**PVP**	**PIP**	**PVAc**	**PPO**	**EC**
Tg (°C)	100	105	147	120	-67	42	211	130
δ_d (MPa$^{1/2}$)	21.3	18.6	19.1	15.5	16.9	19.45	17.9	19
δ_p (MPa$^{1/2}$)	5.8	10.5	7.9	11.7	1.1	10.59	3.1	5.6
δ_h (MPa$^{1/2}$)	4.3	7.5	9.3	8.6	-0.4	5.76	8.5	4.9
Ra with MEH-PPV	4.64	5.60	4.61	10.03	8.43	5.28	4.47	0.60
Ra with PFO	6.27	8.26	7.08	11.54	6.16	8.09	4.21	2.97

[S2] Duong, D. T.; Walker, B.; Lin, J.; Kim, C.; Love, J.; Purushothaman, B.; Anthony, J. E.; Nguyen, T.-Q. Molecular solubility and Hansen solubility parameters for the analysis of phase separation in bulk heterojunctions. *J. Polym. Sci. B Polym. Phys.* **2012**, 50, 1405-1413.

[S3] Hansen, C. M. Hansen solubility parameters: a user's handbook; CRC Press, 2007.

[S4] Sprenger, M.; Walheim, S.; Budkowski, A.; Steiner, U. Hierarchic structure formation in binary and ternary polymer blends. *Interface Sci.* **2003**, 11, 225-235.

[S5] Walheim, S.; Böltau, M.; Mlynek, J.; Krausch, G.; Steiner, U. Structure formation via polymer demixing in spin-cast films. *Macromolecules* **1997**, 30, 4995-5003.

[S6] Cerisier, P.; Porterie, B.; Kaiss, A.; Cordonnier, J. Transport and sedimentation of solid particles in Bénard hexagonal cells. *Eur. Phys. J. E* **2005**, 18, 85-93.

[S7] Camacho, J.; Díez, E.; Díaz, I.; Ovejero, G. Hansen solubility parameter: from polyethylene and poly(vinyl acetate) homopolymers to ethylene–vinyl acetate copolymers. *Polym. Int.* **2017**, 66, 1013-1020.

D. Determination of δΩ, the FWHM of the 0-0 band:

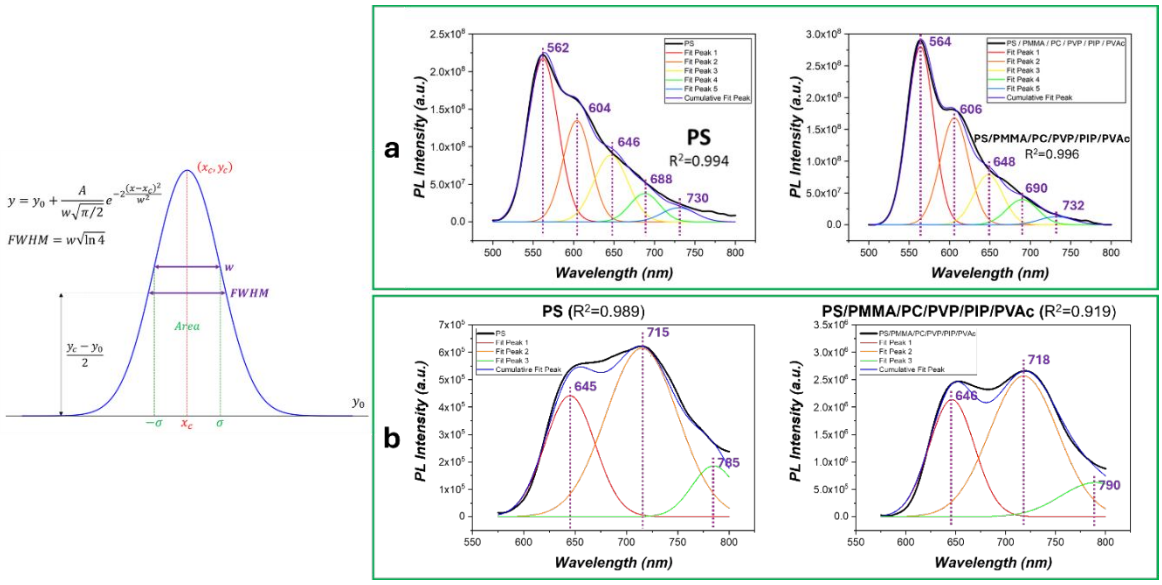

Figure S5. The breadth (δΩ; FWHM) of the PL 0-0 band was determined from the 0-0 peak by fitting the entire spectrum with multiple emissions comprising the 0-0 band and a series of equal-spaced vibronic peaks: (a) for 1 wt.% MEH-PPV and (b) 1 wt.% P3HT-rr, in PS and a ***n*** = 6 matrices (PS/PMMA/PC/PVP/PIP/PVAc).

E. The Luminescence Lifetimes and Quantum Efficiencies:

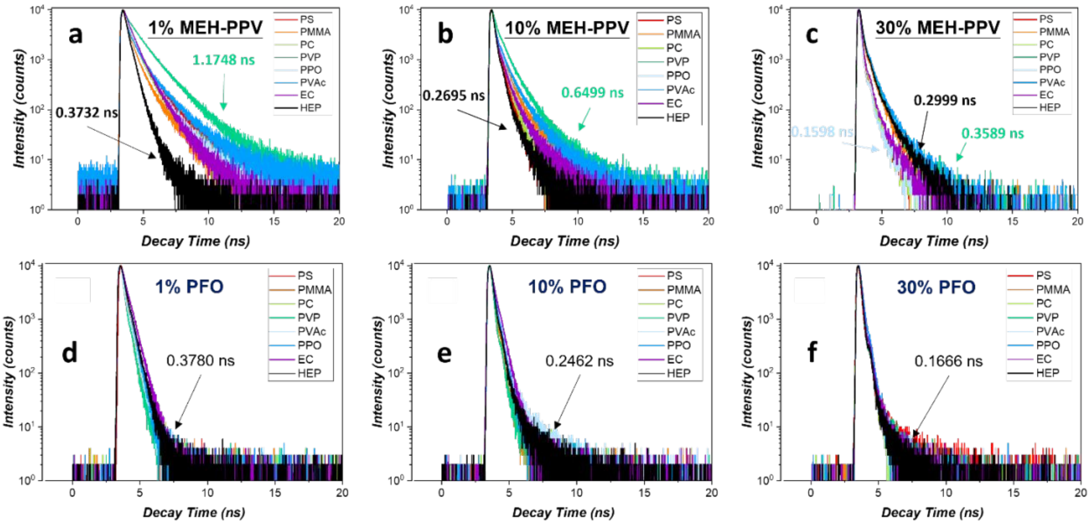

Figure S6. (a-c) The TCSPC spectra of 1, 10, and 30 wt.% MEH-PPV in the different polymer matrices. (d-f) The TCSPC spectra of 1, 10, and 30 wt.% PFO in the different polymer matrices.

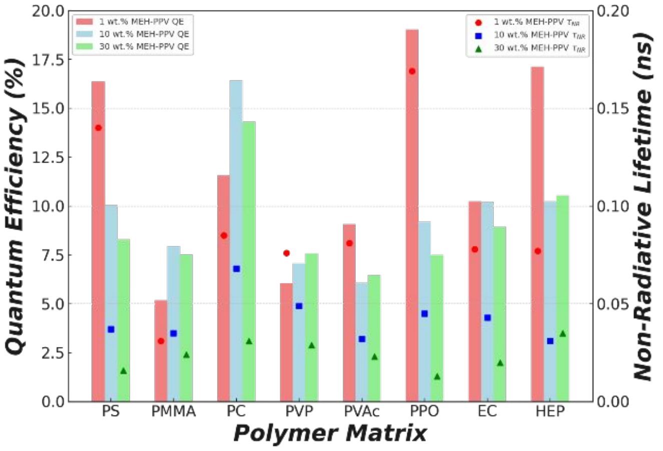

Figure S7. The quantum efficiencies η_R's and the non-radiative lifetime τ_{NR} for MEH-PPV of various concentrations (1, 10, and 30 wt.%) in the various polymer matrices.

Table S2. The radiative (τ_1, τ_2, τ_R) and non-radiative (τ_{NR}) lifetime of 1 wt.% MEH-PPV in different polymer matrices. The luminescent efficiency η_R is acquired from integrating sphere PL. HEP here refers to polymers other than 1 wt.% MEH-PPV is composed of equal proportions.

Polymer	**PS**	**PMMA**	**PC**	**PVP**	**PVAc**	**PPO**	**EC**	**HEP**
τ_1 (ns)	0.51062	0.42911	0.49437	0.87418	0.517	0.53424	0.55154	0.33525
τ_1 percentage (%)	78.16	83.03	81.21	68.54	73.76	76.94	79.80	89.47
τ_2 (ns)	1.456	1.2632	1.308	1.83	1.6356	1.3387	1.2017	0.6952
τ_2 percentage (%)	21.84	16.97	18.79	31.46	26.24	23.06	20.20	10.53
τ_R (ns)	0.7171	0.5707	0.6472	1.1748	0.8106	0.7198	0.6829	0.3732
η_R (%)	16.36	5.17	11.56	6.05	9.08	19.03	10.23	17.13
τ_{NR} (ns)	0.140	0.031	0.085	0.076	0.081	0.169	0.078	0.077

Table S3. The radiative (τ_1, τ_2, τ_R) and non-radiative (τ_{NR}) lifetime of 10 wt.% MEH-PPV in different polymer matrices. The luminescent efficiency η_R is acquired from integrating sphere PL. HEP here refers to polymers other than 10 wt.% MEH-PPV is composed of equal proportions.

Polymer	**PS**	**PMMA**	**PC**	**PVP**	**PVAc**	**PPO**	**EC**	**HEP**
τ_1 (ns)	0.24222	0.24409	0.25961	0.47095	0.30309	0.34634	0.24854	0.19066
τ_1 percentage (%)	76.00	74.01	80.65	76.34	74.38	83.39	77.55	78.48
τ_2 (ns)	0.60245	0.85580	0.70260	1.2272	1.0650	0.9438	0.8185	0.55724
τ_2 percentage (%)	24.00	25.99	19.35	23.66	25.62	16.61	22.45	21.52
τ_R (ns)	0.3287	0.4031	0.3453	0.6499	0.4983	0.4456	0.3765	0.2695
η_R (%)	10.06	7.95	16.43	7.07	6.07	9.20	10.20	10.23
τ_{NR} (ns)	0.037	0.035	0.068	0.049	0.032	0.045	0.043	0.031

Table S4. The radiative (τ_1, τ_2, τ_R) and non-radiative (τ_{NR}) lifetime of 30 wt.% MEH-PPV in different polymer matrices. The luminescent efficiency η_R is acquired from integrating sphere PL. HEP here refers to polymers other than 30 wt.% MEH-PPV is composed of equal proportions.

Polymer	**PS**	**PMMA**	**PC**	**PVP**	**PVAc**	**PPO**	**EC**	**HEP**
τ_1 (ns)	0.1153	0.1632	0.1408	0.2142	0.1960	0.1046	0.1258	0.1893
τ_1 percentage (%)	84.55	75.14	89.35	73.10	77.66	86.89	81.43	79.59
τ_2 (ns)	0.5208	0.6753	0.5737	0.7521	0.7829	0.5263	0.5632	0.7313
τ_2 percentage (%)	15.45	24.86	10.65	26.90	22.34	13.11	18.57	20.41
τ_R (ns)	0.1779	0.2906	0.1869	0.3589	0.3271	0.1598	0.2071	0.2999
η_R (%)	8.30	7.53	14.32	7.58	6.46	7.50	8.94	10.54
τ_{NR} (ns)	0.016	0.024	0.031	0.029	0.023	0.013	0.020	0.035

Table S5. The radiative (τ_R) lifetime of 1, 10, and 30 wt.% PFO in different polymer matrices. HEP here refers to polymers other than PFO that are composed of equal proportions.

Polymer Matrix		**PS**	**PMMA**	**PC**	**PVP**	**PVAc**	**PPO**	**EC**	**HEP**
**1 wt.% PFO**	τ_1 (ns)	0.2846	0.2237	0.3697	0.1873	0.1642	0.3534	0.4923	0.3780
	τ_1 (%)	85.28	61.81	~100	73.90	52.11	~100	~100	~100
	τ_2 (ns)	0.5074	0.4070	-	0.4054	0.4083	-	-	-
	τ_2 (%)	14.72	38.19	-	26.10	47.89	-	-	-
	τ_R (ns)	0.3174	0.2937	0.3697	0.2442	0.2811	0.3534	0.4293	0.3780
**10 wt.% PFO**	τ_1 (ns)	0.1439	0.1791	0.1690	0.1442	0.2595	0.2624	0.2018	0.1944
	τ_1 (%)	66.33	74.81	57.39	83.31	95.61	94.99	26.91	81.33
	τ_2 (ns)	0.3490	0.4343	0.3927	0.4096	0.8568	0.6306	0.3903	0.4723
	τ_2 (%)	33.67	25.19	42.61	16.69	4.39	5.01	73.09	18.67
	τ_R (ns)	0.2130	0.2433	0.2643	0.1885	0.2857	0.2809	0.3396	0.2462
**30 wt.% PFO**	τ_1 (ns)	0.1202	0.1390	0.1298	0.1262	0.1289	0.1755	0.1434	0.1229
	τ_1 (%)	85.07	78.68	80.58	87.88	81.94	77.05	81.19%	82.86
	τ_2 (ns)	0.4146	0.3633	0.3575	0.4403	0.3707	0.3653	0.3862	0.3778
	τ_2 (%)	14.93	21.32	19.42	12.12	18.06	22.95	18.81	17.14
	τ_R (ns)	0.1641	0.1868	0.1740	0.1642	0.1726	0.2191	0.1890	0.1666

F. Photonic Properties of the 1.0 wt.% PFO in the Various Polymer Blends:

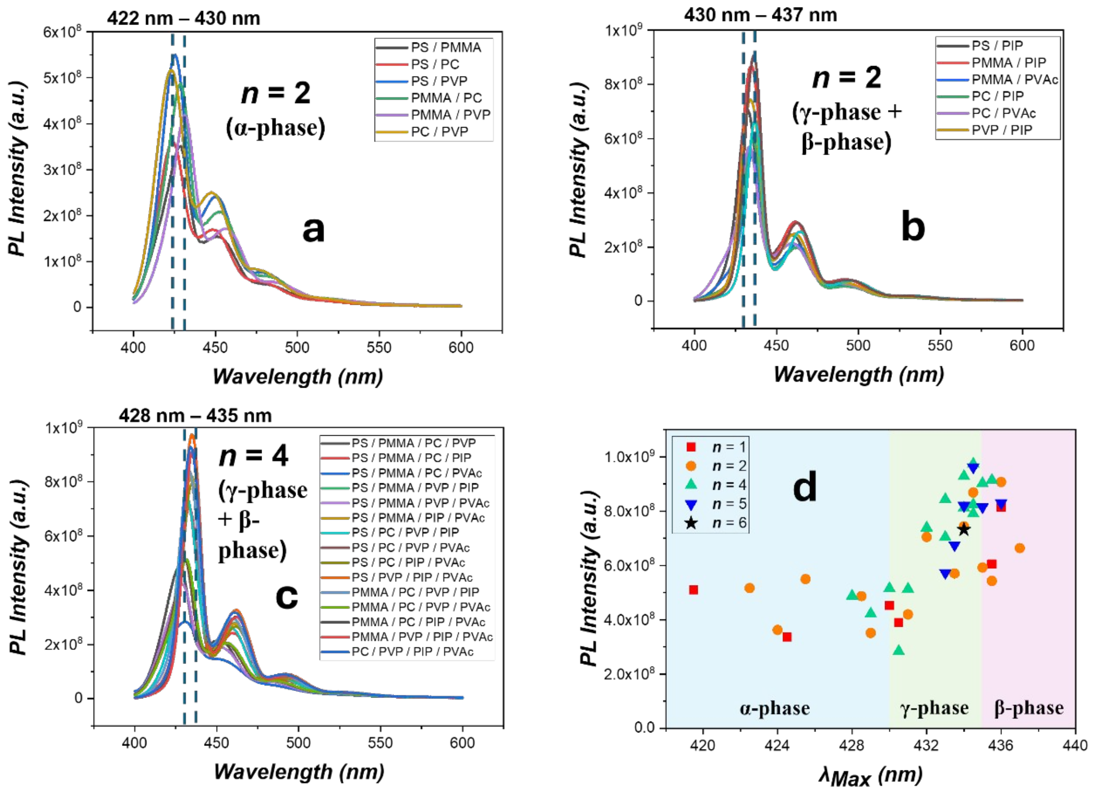

Figure S8. PL spectra of the 1 wt.% PFO in (a) the binary blends (without PIP or PVAc) that show the α-phase, (b) the binary blends (with PIP or PVAc) that show the γ-phase combined with the β-phase, (c) the $\boldsymbol{n}$ = 4 matrices that show the combined γ and β phases, and (d) The PL intensity vs the $\lambda_{\max}$'s of 1 wt.% PFO in the various polymer matrices.

G. Photonic Properties of the 1.0 wt.% P3HT-rr in the Various Polymer Blends:

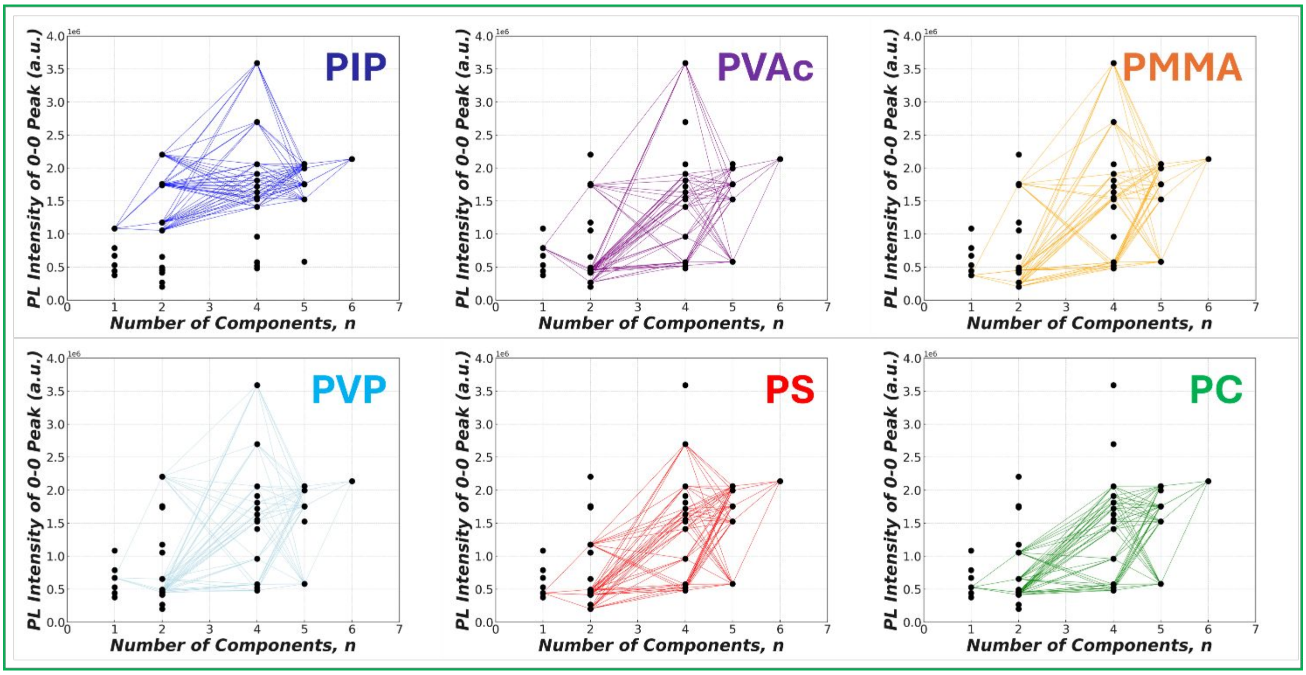

Figure S9. Tracing on the 0-0 band PL intensity of the 1.0 wt.% P3HT-rr for each polymer component *vs*. ***n*** in the polymer matrices.

H. Photonic Properties of Concentrated CP in the Polymer Matrices:

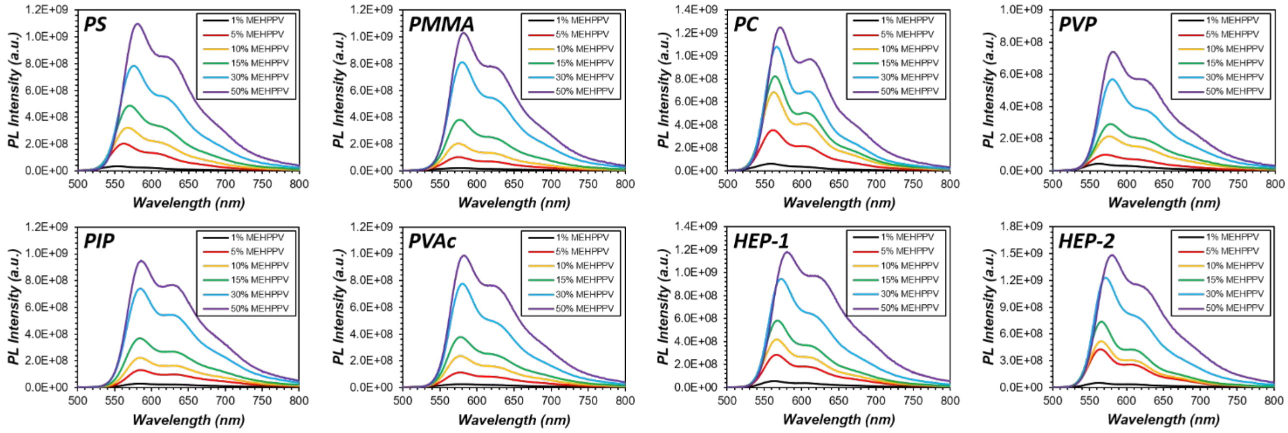

Figure S10. The PL spectra of varied MEH-PPV concentrations from 1 wt.% to 50 wt.% in different polymer matrices. (HEP-1: equal-parted PS, PMMA, PC, PVP, PIP; HEP-2: equal-parted PS, PMMA, PC, PVP, PVAc.)
